# Integrating HIV and mental health interventions to address a global syndemic among men who have sex with men

**DOI:** 10.1016/S2352-3018(22)00076-5

**Published:** 2022-06-21

**Authors:** Don Operario, Shufang Sun, Amiel Nazer Bermudez, Rainier Masa, Sylvia Shangani, Elise van der Elst, Eduard Sanders

**Affiliations:** School of Public Health, Brown University, Providence, RI, USA; School of Public Health, Brown University, Providence, RI, USA; College of Public Health, University of the Philippines Manila, Manila, Philippines; School of Social Work, University of North Carolina Chapel Hill, Chapel Hill, NC, USA; College of Health Sciences, Old Dominion University, Norfolk, VA, USA; KEMRI-Wellcome Trust Research Programme, Kilifi, Kenya; KEMRI-Wellcome Trust Research Programme, Kilifi, Kenya; Nuffield Department of Medicine, University of Oxford, Oxford, UK

## Abstract

In this Series paper, we review evidence on the co-occurring and synergistic epidemics (syndemic) of HIV and mental health problems worldwide among men who have sex with men (MSM). The multilevel determinants of this global syndemic include structural factors that enable stigma, systematic bias, and violence towards MSM across geographical and cultural contexts. Cumulative exposure to these factors over time results in population-level inequities in the burden of HIV infections and mental health problems among MSM. Evidence for this syndemic among MSM is strongest in the USA, Canada, western Europe, and parts of Asia and Latin America, with emerging evidence from sub-Saharan Africa. Integrated interventions are needed to address syndemics of HIV and mental health problems that challenge the wellbeing of MSM populations worldwide, and such interventions should consider various mental health conditions (eg, depression, anxiety, trauma, and suicidality) and their unique expressions and relationships with HIV outcomes depending on cultural contexts. In addition, interventions should identify and intervene with locally relevant structural factors that result in HIV and mental health vulnerabilities among MSM.

## Introduction

In 1981, the first known HIV/AIDS case report described a cluster of unexplained infections among men who have sex with men (MSM) in the USA.^1^ HIV/AIDS has since developed into a global pandemic that affects diverse populations.^2^ However, MSM are a priority population for prevention and treatment interventions worldwide, including in low-income countries and increasingly in sub-Saharan Africa, where MSM have not been historically visible.^3–6^ The 2021 UNAIDS Global AIDS Update reported that the risk of HIV acquisition is 25 times greater among MSM than among heterosexual men.^7^ In 2020, MSM accounted for 75% of new HIV infections in western Europe, the USA, and Canada, 53% in Asia, 46% in Latin America, 21% in the Caribbean, 20% in the Middle East and northern Africa, 16% in eastern Europe and central Asia, 14% in western and central Africa, and 4% in eastern and southern Africa.^7^ However, stigma and discrimination are likely to contribute to reporting bias and underestimates in regions with strong stigma towards MSM.

In the past 40 years, increasing attention has been given to the multidimensional mental health vulnerabilities of MSM, such as depression, anxiety, substance use, and trauma. For example, the prevalence of depression in MSM in the USA is estimated to be 17·2%, which is higher than the overall prevalence for adult men in the USA,^8^ and the risks of post-traumatic stress and substance use are at least two times higher in MSM.^9,10^ A 2017 meta-analysis estimated the pooled lifetime prevalence of suicidal ideation at 25·8% among HIV-positive MSM and 17·0% among HIV-negative MSM.^11^ In the USA, rates of post-traumatic stress are much higher in HIV-positive MSM (up to 74%) than in the general population (8%).^12–14^ Most research into the mental health challenges faced by MSM comes from the USA, Canada, and western Europe,^15,16^ although data are increasingly available from Asia,^17,18^ sub-Saharan Africa,^19–21^ Latin America,^22–24^ and eastern Europe and central Asia.^25–27^

Evidence for the co-occurring epidemics of HIV and mental health problems among MSM align with the concept of a syndemic—the concentration and interaction of these two epidemics in a manner that exacerbates illness, disease, and death within this population.^28^ The strongest evidence for syndemics of HIV and mental health problems among MSM comes from the USA, Canada, western Europe, and some parts of Asia and Latin America, with emerging evidence from sub-Saharan Africa. The application of syndemic framework provides a means to understand the synergies and exacerbated population effects of these complex, multidimensional health conditions. Syndemic framework also underscores the social and structural inequities that marginalise MSM communities across geographical boundaries, limiting opportunities for optimal health among individual MSM.^29^

In this Series paper, we review literature on the multilevel factors that contribute to the global syndemic of HIV and mental health problems among MSM and, on the basis of current evidence, offer recommendations for investment in culturally responsive services to address these synergistic health conditions. We use a scoping approach that considers systematic reviews, relevant intervention trials published since 2016, community surveys, and epidemiological reports on HIV and mental health in MSM populations without geographical restrictions. We did not do a protocol-driven systematic literature search, as our aim was to identify and collate selected findings from relevant illustrative studies.

## Complex determinants of HIV and mental health problems

Conceptual frameworks have described multilevel factors that contribute to the disproportionate burden of HIV and mental health conditions among MSM. Although a comprehensive review is beyond the scope of this Series paper, several conceptual frameworks are useful for guiding the design and implementation of public health interventions for MSM populations (figure, panel 1).

Herek’s sexual prejudice framework provides a foundation for understanding the nature of anti-MSM bias worldwide.^30,31^ Rather than conceptualising this bias as a form of fear towards same-sex sexuality (ie, homophobia), this framework describes sexual prejudice as the active expression of antipathy towards non-heterosexual behaviours and identities.^31^ Sexual prejudice derives jointly from heterosexism (a society’s prioritisation of heteronormative sexual attraction and relationships) and sexual stigma (a society’s collective disparagement of non-heterosexual expressions). Previous reviews have described how these factors have downstream effects on individual-level behavioural and biological risks that contribute to HIV transmission and psychological health.^32^

The minority stress model posits that exposure to sexual prejudice and anti-MSM stigma determine adverse behavioural and physiological health outcomes.^33^ Stigma can occur at multiple levels, including internalised, anticipated, enacted, and structural forms of bias.^34^ Repeated exposure to forms of anti-MSM stigma can produce emotional distress, pressure to conceal one’s identity, social isolation, maladaptive coping, and physiological reactivity. Cross-cultural evidence for the role of minority stress as a determinant of mental health problems is emerging in multiple countries.^35^ Several studies have found links between exposure to minority stress and HIV risk or treatment outcomes among MSM.^36–38^

A life-course epidemiology approach has been used to explain current mental health and HIV status, including HIV-related behaviours, as a function of the accumulation of risks and protective factors throughout a person’s previous life stages.^39^ This approach uses a temporal and social perspective to understand current health patterns, recognising the effect of earlier life events (eg, childhood abuse, peer bullying, and early life trauma) on HIV and mental health outcomes later in life.^40–43^

Originally born out of Black feminist scholarship, intersectionality theory examines the interlocking systems of privilege and oppression that operate at social and structural levels and influence the individual level for people with complex identities that are defined by minoritised race, ethnicity, gender, sexual orientation, and other marginalised statuses.^44^ Identities do not exist in isolation, and MSM with multiple marginalised identities (for example, MSM who are also ethnic minorities in their local context, economically disadvantaged, migrants, sex workers, and members of justice-involved or drug-using subgroups) experience negative effects of intersecting stigmas to a greater extent than do MSM with single marginalised identities. HIV and mental health interventions for MSM should be cognisant of multiple stigmas simultaneously determining health vulnerabilities among individual members of the population.

The health equity promotion model offers a strengths-based perspective to support MSM to reach optimal physical and mental health; the framework is broadly relevant for all sexual-minority and gender-minority populations.^45^ This model considers the role of health-promoting factors, such as individual-level agency and collective forms of advocacy, as mechanisms to challenge the adverse social and psychological processes that result in poor health of MSM. Factors that MSM can use to promote their health vary according to context, culture, and an individual’s position within society. This framework has been used primarily in mental health research with MSM, and applications to HIV have been scarce.

Each of these frameworks aligns with the socio-ecological systems model, which considers multilevel determinants of health for individuals.^46^ Together, these models provide complementary insight into the complex development of syndemics of HIV and mental health problems in MSM, and highlight the shortcomings of individual-level behaviour-change models for HIV prevention that do not account for mental health problems and the upstream determinants of these syndemics.

## Evidence of multilevel determinants of HIV and mental health problems

Studies of the social and structural determinants of HIV and mental health inequities in MSM are most commonly done in the USA, Canada, and western Europe. Reviews of USA-based studies corroborate the role of multilevel stigma and minority stress as determinants of HIV and mental health in MSM.^32,47–49^ For example, self-stigma among MSM in the USA has been associated with emotional distress and HIV testing and diagnosis.^47,50^ A study of MSM in 28 European countries found that structural stigma (eg, discriminatory policies and laws) was associated with pressures to conceal one’s identity and with lower life satisfaction,^51^ and a related study of MSM from 38 European countries found that structural stigma was associated with lower use of HIV-prevention services and higher HIV risk behaviours.^52^ Research involving MSM from 48 countries (mostly in Europe) reported that those who migrated from a country with highly stigmatising laws to countries in which MSM were less stigmatised had a significantly lower risk of depression, suicidality, internalised stigma, and social isolation after migration.^53^ Studies in the USA and Canada have used intersectionality framework to analyse the role of compounding stigmas on health inequities among Black and Latino MSM, who have disproportionately higher HIV risk than White MSM.^54^

Anti-MSM stigmas have been characterised in many parts of Asia, where there is increasing research that describes associations between minority stress, HIV, and mental health.^35,55,56^ For example, a study based in India found that anti-MSM legislation was associated with pressure to conceal one’s sexual identity and was predictive of depressive symptoms.^57^ A study of MSM in Thailand found that lifetime suicidal attempts were associated with experiences of social discrimination, stress, internalised homophobia, and loneliness.^58^ Experiences of minority stress have also been linked with HIV risk behaviour among MSM in China,^59,60^ and with treatment engagement and mental health problems among HIV-positive MSM in China.^61^ A history of discrimination and anticipated stigma in health-service settings have been noted as barriers to engagement with HIV prevention or treatment strategies for MSM in Vietnam, Philippines, and Malaysia.^62–64^ Syndemic analysis has been used to examine health inequities (eg, HIV, mental health, and other behavioural health risks) in MSM in several Asian countries, including Taiwan, Malaysia, and China.^65–67^ To date, intersectionality theory has been used infrequently in research involving MSM in Asia, although increasing attention to social class and migration status among MSM in this region suggests that the use of this theory would be of practical relevance.

Multilevel stigmas towards MSM in sub-Saharan Africa have been documented, including family rejection, peer abuse and bullying, community violence, and anti-MSM policies.^68^ 26 countries in sub-Saharan Africa have legislation that criminalises same-sex behaviour. Anti-MSM stigma and discrimination are linked to poor mental health in studies of MSM in South Africa, Lesotho, Cameroon, and Tanzania.^69–72^ A systematic review of 75 studies assessing HIV testing and engagement among MSM in Africa found that HIV testing and awareness of HIV status were lowest in countries with the most severe levels of structural stigma.^73^ Disclosure of sexual identity was higher in less stigmatising African countries; such disclosure could facilitate engagement in HIV testing and prevention in these countries.^73^ However, that medical environments are safe contexts for disclosure cannot be assumed. A study of MSM in Cameroon, Côte d’Ivoire, Eswatini, Lesotho, and Senegal found that MSM who had disclosed their sexuality to health-care workers were more likely to express fear of health services, avoid health services, face gossiping by health-care workers, and feel mistreated in the health centre.^74^ These findings align with minority stress theory by revealing adverse interpersonal consequences, such as stigma and shame, for MSM who disclose their sexuality in unsupportive medical environments, which can ultimately result in avoidance of and disengagement with health care. Consistent with syndemic framework, studies in several African countries have shown synergistic links between HIV, mental health, and contextual inequalities among MSM.^75–78^ Few studies to date have used intersectionality to analyse health inequities among MSM in Africa, despite recognition of co-occurring stigmas among MSM with multiple marginalised identities in this region.^77,79^

Studies in Latin America corroborate the negative consequences of minority stress for HIV and mental health outcomes. Exposure to anti-MSM stigma is associated with mental health problems among MSM in Chile, Mexico, Brazil, and Peru,^80–83^ and with HIV risk and prevention behaviours among MSM in El Salvador, Belize, and Guatemala.^36,84,85^ A few studies have applied syndemic theory to examine health inequities among MSM in Colombia, Mexico, and Brazil.^86–88^ For example, a study in Mexico found that the presence of a larger number of syndemic conditions (eg, depression, internalised homophobia, violence, and substance use) was associated with greater HIV risk behaviour; however, this association was weaker for men who had disclosed their sexuality to more people, which suggests that openness about sexuality could promote resilience to HIV risks.

A 2020 systematic review^89^ of psychological and behavioural health interventions for sexual-minority and gender-minority populations (published between 2003 and 2019) examined the extent to which these interventions explicitly addressed stigma. This review included 37 published interventions, of which 33 addressed MSM. Most studies were done in the USA (n=24), with fewer studies in Canada (n=4), Australia and New Zealand (n=3), Mexico (n=2), China (n=2), Thailand (n=1), and Senegal (n=1). Internalised stigma (n=24) and enacted stigma (n=21) were the most common foci of these interventions, followed by anticipated stigma (n=12), with few studies addressing structural stigma (n=3).

Another systematic review^90^ in 2020 examined the extent to which mental health interventions for people from sexual minorities integrated principles of inter-sectionality theory. The review identified 43 mental health interventions for people from sexual minorities, of which seven (16%) actively addressed the effects of participants’ interlocking marginalised identities and explicitly used intersectionality theory. Of the interventions that exclusively targeted MSM (n=29), only three (10%) used intersectionality theory. Notably, the majority of mental health interventions for MSM were in the USA (n=21), with three in Europe (the Netherlands, Romania, and Switzerland), two in Canada, and one each in Australia, China, and South Africa.

Ultimately, addressing the social determinants of HIV and mental health inequities faced by MSM will require greater emphasis on community and structural interventions to improve the environments in which MSM live, to enhance access to interventions, to improve the quality of care, and to reduce the barriers to healthy living for all. Interventions that conceptualise mental health and HIV risk among MSM as exclusively individual-level phenomena, without recognising social determinants, could have little effect on improving population-level outcomes. Consequently, if left understudied and unchallenged, multilevel anti-MSM stigmas and sexual minority stress will continue to be major impediments to achieving the UNAIDS 95-95-95 targets for the HIV care cascade by 2030, particularly in settings with severe sexual minority stigma.

## Integrated interventions for MSM to end the global HIV epidemic

Interventions that address the interconnections between mental health and HIV risk, prevention, and treatment outcomes among MSM are necessary to achieve population-level improvements in health. However, health interventions for MSM are frequently designed to prioritise HIV over other health and survival needs. The modest and short-term effects of many HIV-specific interventions for MSM can be enhanced by integrating attention to mental health concerns, which are often personally prioritised by MSM as more immediate and proximal than their sexual health risks.^91^

Some interventions for HIV-negative MSM have targeted both sexual risk behaviours and mental health symptoms. For example, 40 & Forward—a group-based intervention for ageing MSM (40 years and older) who self-report depression, social anxiety, and isolation—was found to be effective in reducing these mental health issues and enhancing self-efficacy regarding condom use.^92^ In response to the high prevalence of HIV risk among MSM who were sexually abused in childhood, an intervention programme in the USA provided evidence for the integration of cognitive behavioural therapy for trauma and self-care as a means to reduce HIV risk and symptoms of post-traumatic stress disorder.^93^ Another intervention with HIV-negative MSM in India compared resilience-focused psychosocial group and individual counselling with standard-of-care as a control, and found larger reductions in condomless sex and depressive symptoms and larger improvements in self-esteem in the intervention group than in the control group, from baseline to 12-month follow-up.^94^ A transdiagnostic cognitive behavioural therapy programme called ESTEEM targeted young MSM in the USA who had reported recent condomless sex and psychological distress; the programme reduced depression, alcohol use, and condomless sex compared with a waitlist control.^95^ Adaptation and evaluation of this intervention for MSM in Romania found an increase in HIV knowledge, recent HIV testing, and self-efficacy regarding condom use, and a reduction in heavy alcohol use and symptoms of mental health problems among programme recipients.^96^ The programme has also been culturally adapted for delivery to MSM in China.^97^

Mental health interventions for MSM living with HIV have focused on various issues facing this population, such as psychological wellbeing, substance use, sexual health, and HIV care. Examples of interventions include cognitive behavioural therapy for HIV-positive MSM with psychological distress in China,^98^ and in the USA, the use of a unified protocol for MSM living with HIV with sexual compulsivity issues,^99^ group-based, mindfulness-based stress reduction for gay men living with HIV,^100^ and tailored mindfulness-based intervention for MSM living with HIV who use methamphetamine.^101^ Integrated interventions that address intersectional issues (eg, racial discrimination and sexual stigma) and mitigate structural issues (eg, unemployment) among MSM are also being developed.^102,103^ This growing body of research highlights the potential of mental health interventions to improve sexual health, reduce HIV risk, and support MSM living with HIV as they navigate HIV prevention and health-care systems.

The current body of literature on integrated HIV and mental health interventions has several limitations. First, most of the research has been done in the USA, which raises concerns about generalisability to settings in which sexuality and mental health are shaped by unique cultural factors and health-service systems are very different. Much has been written about the need to decolonise public health and psychological interventions.^104,105^ In this regard, mental health services should be recognised as culturally encapsulated healing practices that have been generated typically through an individualistic cultural model (and financial model) of psychological health services based in western European or US settings.^106^ The damaging history and legacies of such mental health models related to same-sex sexuality (ie, the American Psychiatric Association categorising same-sex attraction as a mental illness until 1973), which have shaped understanding of sexual orientation in many contexts in other regions, should also be acknowledged.^107^ The same-sex sexual identities of men are also shaped by cultural, historic, neo-colonial, political, and economic factors in specific regions. Researchers and stakeholders should understand the specific context that shapes the identities, lived experiences, and health outcomes of MSM, and develop locally relevant and responsive programmes to minimise potential harm. For example, in high-stigma contexts, identity concealment is a common and adaptive coping strategy.^56^ Short-term interventions (eg, time-limited therapy), which are often practised in the psychiatric health-care system in the USA, might provide only a very limited approach to address mental health issues for MSM in other countries. Second, the mental health symptoms and experiences of MSM addressed by the current research are heterogenous. Expressions of mental health problems are likely to be context-dependent, and the relationship of these symptoms with HIV risk could vary depending on local cultural systems. Third, few structural interventions tailored for MSM address factors linked to poor mental health such as incarceration and human rights issues, economic concerns, housing and workplace discrimination, relationship validation, and marriage laws. Finally, recognition that community-based, non-governmental organisations in many parts of the world already provide locally developed, integrated approaches to address HIV and mental health among MSM is important. For example, the Humsufar Trust in Mumbai, India provides programmes that address HIV, mental health, social support, and legal advocacy for MSM and other people from sexual-minority and gender-minority populations, including young people living with HIV. Love Yourself, based in Manila, Philippines, is a non-governmental organisation that serves MSM and people from other minority populations by offering HIV testing, prevention, and treatment support services, and peer-based counselling to address mental health needs. In Cape Town, South Africa, the Triangle Project delivers HIV prevention, testing and treatment referrals, and mental health support services, legal advocacy, and substance-use harm-reduction services to MSM and other sexual-minority and gender-minority populations. In accordance with syndemic and inter-sectionality frameworks, organisations such as these typically do not address health conditions in isolation or regard clients solely on the basis of their sexual identity; furthermore, commensurate with the health equity promotion model, such organisations acknowledge the strengths and assets within local client communities and advocate for social change. However, many community organisations that serve MSM and other sexual-minority and gender-minority populations are underfunded or receive funds that are earmarked to address single health conditions (eg, exclusively HIV services). Partnerships and investment in these and other frontline community organisations is a crucial component of global efforts to improve HIV and mental health outcomes among MSM and other sexual-minority and gender-minority populations (panel 2).

## Structural interventions to promote societal change and improve health equity

As of 2020, same-sex behaviours were criminalised in 71 countries worldwide. Policing of MSM reflects the historical and contemporary social construction of MSM populations as entities to be surveilled and restrained. State-sanctioned stigma impairs sexual and mental health of MSM, and their use of mental health care, HIV prevention and treatment, and other health services.^120^ Similarly, people from sexual minorities in many global settings have been historically pathologised and medicalised as person-objects needing treatment. These characterisations of the lives of MSM have resulted in both formal and social policing, and contribute to the disproportionate presence of men from sexual minorities in secure settings such as jails, prisons, hospitals, juvenile detention, and immigration detention facilities.^121^ Decriminalisation of same-sex behaviours will increase the ability of MSM to be open and not fearful about their sexuality and identities, which is essential for optimising health and use of mental health care and comprehensive HIV prevention and treatment. The historic 2016 vote by the UN Human Rights Council to adopt a resolution protecting MSM and other sexual and gender minorities against violence and discrimination provides a global-level framework to support national-level human rights advocacy efforts.

Discrimination and violence towards MSM often begins in childhood in the forms of family rejection, peer harassment and bullying, and other types of violence including sexual victimisation. This cumulative experience of stigma increases the risk of mental distress and maladaptive coping processes such as substance use.^33,34^ Systematic efforts are needed to disrupt societal patterns of abuse towards children who are perceived as non-heteronormative, and to provide support to children with diverse gender expressions in schools and community settings. Such efforts can reduce vulnerability to sexual, mental, and behavioural health risks later in life.

Initiatives that affirm and legitimise same-sex relationships can transform the sexual and mental health of MSM and other sexual minorities. Evidence supports the mental health benefits for sexual minorities after the passage of marriage-equality laws.^122^ Relationship-strengthening interventions for MSM are associated with lower HIV transmission risk and improved HIV treatment outcomes for MSM living with HIV.^123^ However, evidence indicates that social and political debates about marriage equality are sources of minority stress for individuals from sexual minorities, and thereby contribute to mental health problems.^124^ Conceiving of marriage-equality legislation in many countries is difficult, owing to local histories and belief systems. Culturally aligned approaches are needed to conceptualise opportunities for relationship strengthening and affirmation programmes for same-sex partnerships in countries with highly conservative, heteronormative values.

Recognition and investment in multilevel stakeholders within the fields of public health, biomedicine, and policy are essential to address structural-level aims and to tackle the syndemic of HIV and mental health problems for MSM. Community-based organisations and grassroots initiatives can advocate for policy changes to increase social, legal, and economic equity for and reduce discrimination against sexual minorities. Health providers and health systems can enact more inclusive approaches for MSM and people from other sexual minorities who are seeking sexual, mental, and behavioural health interventions. Transformations within health education institutions and professional training programmes are needed to prepare providers to deliver non-biased and effective services to MSM and other patients from sexual minorities. At all levels of change, individual professionals and organisational systems should acknowledge historic biases and inequitable practices that have disadvantaged MSM and individuals from other sexual minorities who are seeking services and have perpetuated HIV and mental health vulnerabilities. However, interventions should recognise the realities of local context, infrastructure, and epidemiology. MSM-centered initiatives might not resonate with local priorities in settings with generalised high-prevalence HIV epidemics, owing to overwhelming demands at the population level. At a minimum, this Series paper underscores the need for integrated HIV and mental health policies and interventions worldwide to be inclusive of MSM through responsive, engaged, and community-empowering practices.

## Conclusion and future directions

We propose that prioritising the promotion of mental health among MSM is a public health strategy that could help to end the global HIV epidemic. Improving mental health in and of itself is an under-recognised public health priority for MSM populations worldwide. Integrated intervention approaches are needed. We provide several key considerations on the basis of our review (panel 3). First, psychological care is often prioritised for only part of the population (eg, individuals who meet criteria for mental disorders and who have access to psychological and economic resources), rather than considered a basic need for all individuals. Consequently, mental health services are deprioritised and typically decoupled from other health services such as HIV prevention and treatment. The integration of mental health interventions and HIV prevention and care services can happen on various levels, such as a brief mental health assessment and relevant referrals at HIV testing and pre-exposure prophylaxis sites, the inclusion of relevant mental and relational health content as part of sexual health promotion programmes, and the formation and provision of support groups for MSM in schools, workplaces, LGBTQ-related community organisations, and HIV care clinics. Stakeholders in HIV prevention and care services can work with psychological professionals to create more comprehensive systems of care that address mental health as a key target. Second, indigenous, local approaches to mental health interventions should be understood and affirmed. Engaging local MSM communities in the process of intervention development and roll-out might be beneficial to harness resilience factors and effective coping strategies within the local community. In high-prevalence settings, local mental health, HIV prevention and treatment, and stigma-reduction programmes for general populations can be adapted to include MSM in those settings. Third, competent service providers skilled in mental health care for MSM are required. Mental health is a multidimensional construct, requiring sensitivity to unique expressions and symptoms that might vary across contexts and pose differing challenges for HIV prevention and risk. In highly stigmatising and resource-limited settings, targeting common mental health elements (ie, emotional, cognitive, interpersonal, and behavioural components) and training lay providers have proven to be effective approaches.^125^ Furthermore, training physicians, counsellors, and HIV service providers in the care of MSM can encourage MSM to seek psychological help and to address the intersectional stigmas of HIV risk and disease, MSM identity, and mental health. Finally, efforts to acknowledge the contributions of locally developed approaches to promoting health equity for MSM populations in diverse cultural contexts should be more commonly practised. Such efforts are likely to yield innovative solutions for HIV and mental health inequities, which can diversify the range of practices to support the health of MSM populations and contribute to the decolonisation of public health across and within global contexts.

## Figures and Tables

**Figure:**
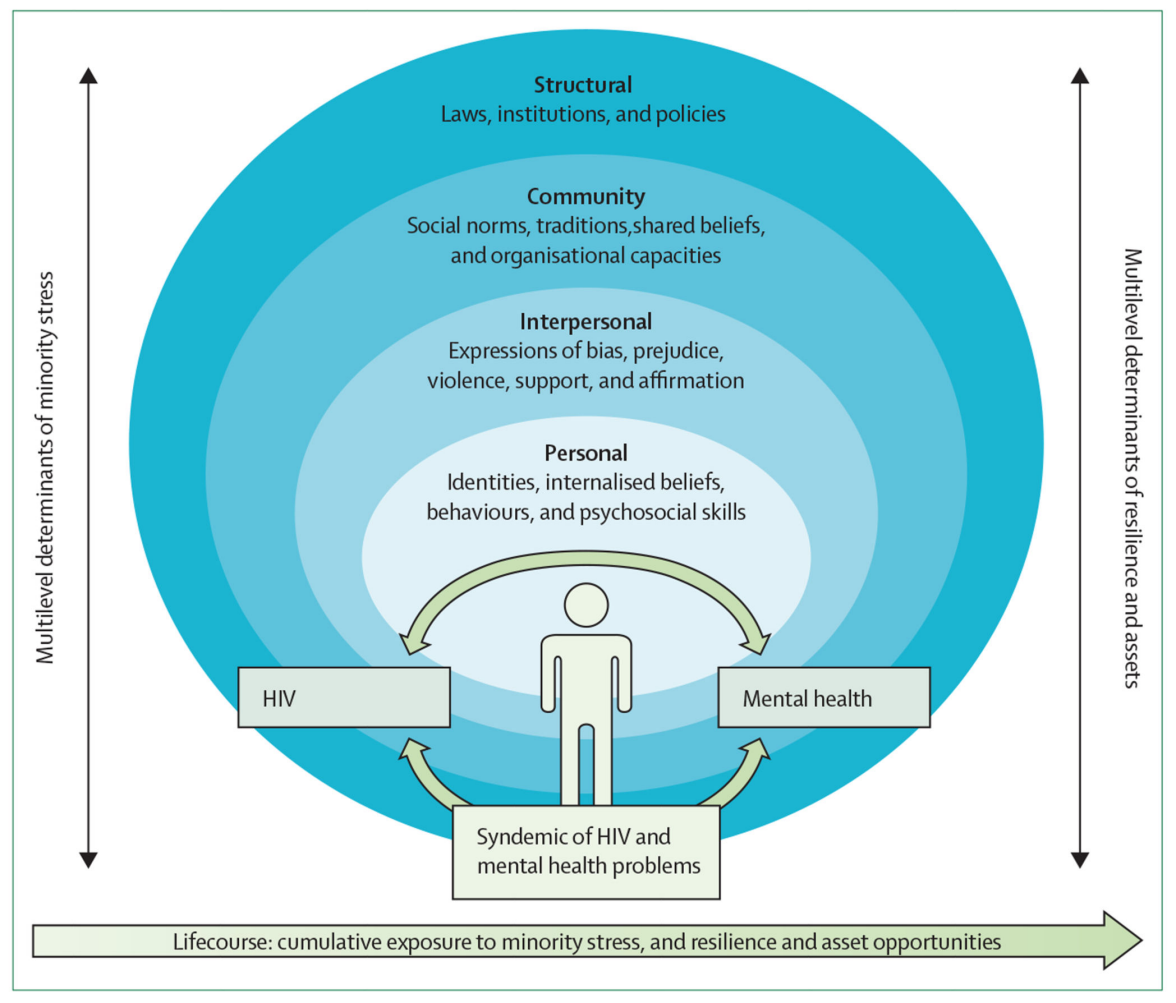
Multilevel processes contributing to syndemics of HIV and mental health problems in men who have sex with men The schematic is based on selected conceptual frameworks with aetiological examples from literature.

## Key messages

- There is strong evidence for a global syndemic of mental health problems and HIV among men who have sex with men (MSM) populations worldwide
- Anti-MSM stigma contributes to disproportionate mental health problems and HIV transmission in this population
- Improving access and quality of mental health support services for MSM worldwide is essential to end the HIV epidemic
- Structural determinants of mental health problems and HIV for MSM present complex challenges and require multilevel public health interventions
- Efforts are needed to expand the professional and paraprofessional mental health workforce, invest in civil society organisations that support MSM, and reform policies to enable full societal inclusion and basic rights for MSM worldwide

## Panel 1 Selected conceptual frameworks to understand and address global syndemics of HIV and mental health problems in MSM

### Syndemic theory

Synergistic and mutually reinforcing HIV and mental health epidemics are concentrated in communities of men who have sex with men (MSM), derive from social and structural inequities, and bring about exacerbated illness, disease, and death in MSM populations.

### Sexual prejudice framework

Anti-MSM bias reflects negative regard towards non-heterosexual behaviours and identities, and arises from social factors including heteronormativity and sexual stigma.

### Minority stress theory

Exposure to chronic distal and proximal stress due to anti-MSM stigma evokes psychological and physiological reactions at the individual level, resulting in mental health and behavioural health vulnerabilities.

### Lifecourse approach

Health status among individual MSM is determined by cumulative earlier life experiences including exposure to bullying, abuse, emotional distress, and rejection due to anti-MSM bias.

### Intersectionality theory

Individuals can have multiple identities, each with distinct histories in terms of power and inequality. These identities converge at the individual level, shaping health, human experience, and access to resources.

### Health equity promotion model

Emphasis on resilience, assets, and agency of MSM to maximise health and wellness, while recognising unequal opportunities and constraints related to intersectional identities and social contexts.

### Socioecological model

Individual, interpersonal, community, and societal-level factors lead to mental health and HIV risk, such as internalised stigma, domestic violence, school bullying, and anti-LGBTQ laws.

## Panel 2 Case study—a transformative HIV research partnership with MSM in coastal Kenya

Research with men who have sex with men (MSM) in coastal Kenya started in 2005 when participants joined an HIV vaccine feasibility cohort study in Mtwapa. The initial studies conducted at the Kenya Medical Research Institute (KEMRI) were among the first on the African continent to show evidence of high HIV incidence among MSM, with HIV incidence rates before the availability of pre-exposure prophylaxis estimated at 8·6 cases per 100 person years.^108^ Research-based services—including HIV testing and counselling, treatment for sexually transmitted infections, MSM peer-support groups, and referrals to community advocacy groups—were provided to MSM who participated in the programme. Integration of social support and community links with the research were the mainstay of the project and contributed to its success.

In 2010, the project site was shut down by members of the local community, who were reacting to rumours that the clinic research site was initiating young men to homosexuality and into same-sex marriages—both of which are considered taboo and are criminalised in Kenya.^109^ A documentary by Sorious Samura, named Africa’s Last Taboo, shows original footage of the event, with large groups congregating outside the clinic, resulting in the temporary shutdown of services and arrest of research volunteers. One KEMRI participant in the programme, who was interviewed in the documentary, says that he would have been set on fire as community members poured paraffin on him and planned to ignite him, and only a miracle saved him.

Violence towards MSM is associated with HIV risk. During a study done between 2005 and 2014 in Mtwapa, the incidence of rape for MSM was 3·9 events per 100 person years.^110^ Physical and verbal assault were also common. In two studies assessing mental health and substance use challenges among MSM in Kenya, many reported childhood abuse (77%), symptoms of depression (31% of participants had Patient Health Questionnaire-9 scores of 10 or more), hazardous alcohol use (41%), or problematic substance use (51%).^111,112^

In response to the clinic shutdown in 2010, the research programme initiated a sensitisation programme for community stakeholders, religious leaders, health-care providers, and government officials.^113–115^ Sensitisation programmes were delivered in collaboration with representatives from local LGBT organisations. The theme of these sensitisations was “facing our fears”, and fears were mitigated through respectful listening to all stakeholders involved. Evaluation in 2013 indicated long-term positive effects on sensitisation programme participants.

Research with MSM continued uninterrupted for another 10 years, until complications related to the COVID pandemic later closed services. Increasingly, research was done in collaboration with LGBT groups and peer mobilisers, including an intervention trial that aimed to improve adherence to antiretroviral therapy among MSM,^116^ a partner notification study that aimed to identify new HIV infections among MSM,^117^ an intermittent pre-exposure prophylaxis study,^118^ and the first known study to suggest that anti-MSM prejudice can be reduced among Kenyan health-care providers after sensitivity training.^119^ Research with MSM was enabled through the strong support for vulnerable populations, the non-judgemental attitudes of research programme staff, and a strong community stakeholder engagement programme.

## Panel 3 Recommendations to address syndemics of HIV and mental health problems in men who have sex with men

- Prioritise mental health as a foundation for HIV prevention and treatment programmes, and for other public health programmes for men who have sex with men (MSM)
- Acknowledge diversity within MSM communities and the presence of multiple stigmas related to intersectional identities
- Develop, adapt, and implement brief integrated interventions that are relevant to the social context of HIV and mental health challenges in local settings
- Recognise indigenous approaches to support mental health in local contexts
- Invest in local community-based organisations that provide holistic health, social, and advocacy services to sexual-minority and gender-minority populations
- Address childhood forms of anti-MSM bias through family, community, and school-based interventions
- Support and affirm family, peer, and intimate relationships for MSM
- Train health and social-service providers in delivery of inclusive programmes for MSM
- Improve structural and legal systems for supporting the rights of people from sexual or gender minorities

## Search strategy and selection criteria

We used a scoping approach that considered systematic reviews, recent relevant intervention trials, community surveys, and epidemiological reports on HIV and mental health in populations of men who have sex with men (MSM). We searched PubMed and PsycINFO using relevant keywords (eg, “MSM”, “gay”, “homosexual*”, “mental health”, “HIV”, and “intervention”) for relevant reports published in English from database inception until Dec 1, 2021, without restriction by geography.
